# Revisiting the roles of VHR/DUSP3 phosphatase in human diseases

**DOI:** 10.6061/clinics/2018/e466s

**Published:** 2018-08-28

**Authors:** Lilian Cristina Russo, Jéssica Oliveira Farias, Pault Yeison Minaya Ferruzo, Lucas Falcão Monteiro, Fábio Luís Forti

**Affiliations:** Departamento de Bioquímica, Instituto de Quimica, Universidade de Sao Paulo, Sao Paulo, SP, BR

**Keywords:** Protein tyrosine phosphatase (PTP), Vaccinia H1-related phosphatase (VHR), Dual-specificity phosphatase 3 (DUSP3), Mitogen-activated protein kinase (MAPK)

## Abstract

Protein tyrosine phosphatases have long been considered key regulators of biological processes and are therefore implicated in the origins of various human diseases. Heterozygosity, mutations, deletions, and the complete loss of some of these enzymes have been reported to cause neurodegenerative diseases, autoimmune syndromes, genetic disorders, metabolic diseases, cancers, and many other physiological imbalances. Vaccinia H1-related phosphatase, also known as dual-specificity phosphatase 3, is a protein tyrosine phosphatase enzyme that regulates the phosphorylation of the mitogen-activated protein kinase signaling pathway, a central mediator of a diversity of biological responses. It has been suggested that vaccinia H1-related phosphatase can act as a tumor suppressor or tumor-promoting phosphatase in different cancers. Furthermore, emerging evidence suggests that this enzyme has many other biological functions, such as roles in immune responses, thrombosis, hemostasis, angiogenesis, and genomic stability, and this broad spectrum of vaccinia H1-related phosphatase activity is likely the result of its diversity of substrates. Hence, fully identifying and characterizing these substrate-phosphatase interactions will facilitate the identification of pharmacological inhibitors of vaccinia H1-related phosphatase that can be evaluated in clinical trials. In this review, we describe the biological processes mediated by vaccinia H1-related phosphatase, especially those related to genomic stability. We also focus on validated substrates and signaling circuitry with clinical relevance in human diseases, particularly oncogenesis.

## Protein Tyrosine Phosphatases, Dual-Specificity Phosphatases, and VHR/DUSP3

Protein tyrosine phosphatases (PTPs) are highly catalytically active enzymes that counteract the actions of protein tyrosine kinases (PTKs). This pair of families has different evolutionary origins. PTPs are organized into four classes (I to IV) based on the presence of either a cysteine or an aspartate in the catalytic site 1-3.

Class I PTPs are subdivided into classical tyrosine phosphatases and non-classical or dual-specificity tyrosine phosphatases (DUSPs), the latter of which are a less common group of enzymes that dephosphorylate both tyrosine and serine/threonine residues 1. DUSPs comprise the largest (61 proteins) and most diversified group of non-classical PTPs. A highly conserved consensus or signature sequence, HC(X)_5_R, is present in the catalytic domain of both classical and non-classical tyrosine phosphatases 1,4. The catalytic mechanisms by which PTPs and DUSPs exert their effects are quite evolutionarily conserved and involve substrate hydrolysis and the formation of a stable phosphoryl-cysteine intermediate 5-7.

The atypical dual-specificity phosphatases (ADUSPs) include 19 proteins and were discovered in the last 10 years. Most of these proteins are small (<27 kDa and/or <250 aa), and because they have the ability to dephosphorylate Tyr and Ser/Thr residues, they can act on substrates, such as mRNAs, lipids, and glycogen 5,8.

ADUSPs possess the consensus catalytic domain of PTPs and typical DUSPs, and this domain is responsible for their phosphatase activity. However, ADUSPs also possess regulatory and/or recognition sequences at their C- and N-termini, and these surround the catalytic active site 9,10. ADUSPs do not have the CH2 (Cdc25 homology 2) domain that is normally associated with typical DUSPs 5,8 and is responsible for their specificity for mitogen-activated protein kinases (MAPKs) 8,11,12. However, even without these recognition sequences, some ADUSPs are capable of dephosphorylating ERK, JNK, and p38 5,8.

This is the case for Vaccinia H1-related phosphatase (VHR) or dual-specificity phosphatase 3 (DUSP3), which was first identified in higher eukaryotes and was the first phosphatase to be crystallized 13,14. It weighs approximately 21 kDa and contains 185 residues, including PTP domains with the HC(X)5R consensus sequence. The VHR crystal structure includes a catalytic site that is 6 Å deep, which is shallower than all other classic PTPs (9 Å deep), and this may explain the substrate diversity observed among this enzyme 5,13,15. VHR is constitutively active, is widely expressed in several tissues, and can localize to either the nucleus or cytosol, with its localization important to its various functions 16,17.

Among the biological roles attributed to VHR (discussed later) is its ability to control the cell cycle, which affects cell proliferation. This makes the study of VHR important for developing therapeutic strategies for different types of cancers. VHR behaves similar to a MAPK phosphatase (MKP or a typical DUSP) in that it can dephosphorylate ERK, JNK, and p38 16,18-22 via a CH2-independent catalytic mechanism. VHR also displays increased catalytic activity in the presence of the serine/threonine kinase VRK3 and the tyrosine kinases Zap70 and Tyk2, all of which bind and/or chemically modify VHR to enhance its activity 19,22,23. These VHR-related regulatory mechanisms warrant further investigation as a potential therapeutic targets for controlling tumorigenesis 5,12.

In addition to MAPKs, other substrates have recently been shown to be targets of VHR. These include the following: 1) the ErbB receptor of EGF2 in non-small cell lung cancers 24, 2) signal transducer and activator of transcription 5 (STAT5) in immune cells stimulated by cytokines and/or growth factors 19, and 3) focal adhesion kinase (FAK), with which VHR plays a role in the formation and disassembly of focal adhesions 25. Furthermore, VHR has been found to have a new MAPK substrate-independent biological function that affects the maintenance of genomic stability. Some authors 26,27 recently suggested that many different nuclear proteins, such as NBS1, NPM, NUCL, hnRNP C1/C2, and ATM/ATR, that are directly or indirectly involved in different steps of the DNA damage response and DNA repair pathways are very likely to be substrates of VHR. These new putative protein targets and functions of VHR will be discussed in the following sections. We argue that this dual phosphatase constitutes a potential candidate for pharmacological targeting in clinical trials.

## Biological functions of VHR/DUSP3 and its association with human diseases

A high percentage of human cancers originate from one or more activating mutations and/or genomic aberrations in members of canonical signaling pathways, such as KRAS and EGFR, that specifically activate the downstream MAPK pathway 28. Furthermore, since VHR dephosphorylates MAPK and growth factor receptors (i.e., HER2) 17,24, VHR is relevant to the study of many types of cancers in which the activities of these signaling pathways are altered. For example, cervical cancer cell lines deficient in VHR arrest at the G1-S and G2-M cell cycle transitions and therefore show massively inhibited cell proliferation followed by initial signs of senescence 17,18.

MAPK activation plays a growth-promoting role during the early G1 phase of the cell cycle 17. Rahmouni et al. 17 showed that differential VHR expression is coupled with cell cycle transitions (i.e., high in the S, G2, and M phases) and may regulate MAPK activity. They also showed that VHR is expressed at low levels in early G1 phase, during which cyclin D1 expression is required for cell cycle progression 29, and that VHR is overexpressed in many types of cancers 30,31. For example, in breast cancers, cyclin D1 expression was found to be downregulated via a variety of mechanisms 32,33. One of these mechanisms involves the VHR-mediated overexpression of BRCA1-IRIS 34,35, which is responsible for Cyclin D1 overexpression and the induction of cell proliferation. Additionally, overexpressing at least one of the components of the BRCA1-IRIS/EGFR/ErbB2 axis reduced VHR expression in normal and tumor breast cell lines, suggesting the development of more aggressive endocrine-resistant breast cancer phenotypes. Therefore, restoring VHR expression could potentially rescue cyclin D1 overexpression-dependent cellular transformation 35.

In non-small cell lung cancer (NSCLC) tissues, VHR expression and its activity against MAPKs were lower than those observed in normal lung tissues, while VHR activity against the ErbB2 receptor, which also regulates epithelial cell growth, was increased 24. Wang et al. 24 demonstrated that VHR can dephosphorylate a specific EGFR isoform in cell lines. The EGFR-VHR interaction is weak, and the binding of the EGF ligand to its receptor makes this interaction even weaker. However, overexpressing VHR reduced the phosphorylation of EGFR at Tyr-992 and made cells less responsive to EGF.

VHR overexpression suppressed cell proliferation in two- or three-dimensional cultures of H1299 cells and reduced the tumor sizes of xenografts. In addition, the mRNA and protein levels of VHR were markedly lower in tumor tissues than in adjacent healthy tissues in patients with non-small cell lung cancers 24. Thus, decreasing VHR expression might contribute to the initiation of lung cancer pathogenesis. Wagner et al. proposed that the onset of lung tumorigenesis in NSCLC involves the enhancement of ERK1/2 signaling as a result of the epigenetic downregulation of VHR by KDM2A demethylase 36. Some histone methylation modifiers, such as KDM2A, are overexpressed in NSCLC and are important for the tumorigenicity, proliferation, and invasiveness of these cells 37. Depleting KDM2A reduced the number of cells in S phase, inhibited their ability to form colonies in soft agar, and, most interestingly, upregulated (by up to 9-fold) the mRNA and protein expression levels of VHR. These data suggest that the epigenetic repression of VHR activates the downstream effects of ERK1/2 signal-mediated proliferation, contributing to the invasive characteristics of NSCLC.

Studies have correlated VHR overexpression with the onset or development of carcinogenic phenotypes. For example, VHR levels were higher in human HeLa, SiHa, CaSki, C33, and HT3 cell lines, which are derived from cervical tissues and consist of epithelial cells representing various grades of squamous carcinomas and adenocarcinomas, than in primary cells and normal tissues 38. In cervical cancer tissues, VHR is primarily enriched in nuclei. However, in tumor cell lines, VHR is localized in both the cytoplasm and nucleus, whereas in normal primary cells, it is exclusively cytosolic 38. The enrichment of VHR in cell lines and cervical cancer tissues is not due to increased gene or protein expression or the stabilization of its mRNA. Instead, it cause by an increase in the post-translational stabilization of the VHR protein 38.

VHR is also enriched in prostate cancer tissues 39. In normal prostate epithelial cells, androgen withdrawal led to decreased cell proliferation, increased apoptosis, and atrophy, whereas tumor prostate epithelial cells overcame these deficiencies by shifting to an androgen-independent state 40,41. When androgen-responsive prostate cancer cell lines were treated with synthetic androgens, various MKPs become overexpressed, including VHR. This changed was correlated with the systematic dysregulation of MAPK signaling observed during prostate carcinogenesis 42, as was observed in LNCaP cells treated with TPA/thapsigargin plus R1881. This dysregulation decreased JNK phosphorylation but did not affect pERK expression levels 43,44. Androgen-sensitive grafts expressing VHR showed increased resistance to castration-induced apoptosis, and tumor regression was inversely correlated with VHR expression 39. One molecular mechanism that has been suggested for inhibiting prostate cancer progression is based on the finding that VHR knockdown led to JNK activation and apoptosis, suggesting potential therapeutic applications aimed at targeting this phosphatase.

Finally, VHR is also overexpressed in dysplastic nevi (DN), which are benign melanocytic tumors with a hyper-proliferative phenotype 45. Very recently, VHR was found to interact with and dephosphorylate FAK in H1299 cells and MEF/lung epithelial cells. In VHR-null mice, these cells were more migratory, and VHR overexpression decreased pFAK levels and inhibited cell migration 25. The enhanced motility phenotype observed in these cells supports the hypothesis that the VHR phosphatase plays important roles in the development of metastatic tumors.

It seems that with regard for the mechanisms underlying tumorigenesis, this dual phosphatase plays two opposing functions. Specifically, VHR knockdown inhibits the proliferation and invasiveness of cancer cell lines, in accordance with oncogene/oncoprotein models. However, in some cellular contexts, VHR downregulation triggers hyper-proliferation, migration and invasiveness in these cells. Thus, it can also be considered a tumor suppressor 46.

Another VHR function associated with tumor onset, establishment, growth, and maintenance is the regulation of angiogenesis, which is a crucial step during tumorigenesis. As shown previously in different types of cancers, endothelial cells express higher than normal levels of VHR and other MKPs, and this alteration was found to contribute to MAPK-independent PKC-mediated tubulogenesis *in vivo*. In the same FGF2-stimulated cells, VHR knockdown blocked angiogenic sprouting. Finally, in different *in vivo* and *ex vivo* models of VHR^-/-^ mice, neovascularization was clearly decreased 47.

Genomic instability is characteristic of a broad spectrum of cancers, and genomic alterations can occur during any cell division. These alterations or instabilities are minimized by four major mechanisms: high-fidelity DNA replication during S phase, precise chromosome segregation in M phase, accurate and error-free repair of DNA damage, and a cell cycle progression that is coordinated by cell cycle checkpoints 48. Therefore, a disruption in any step in one or more of these four mechanisms can lead to genomic instability and contribute to cancer development.

In terms of MAPK signaling, with MAPKs the best characterized substrates of VHR, over-activating ERK1/2 induced multipolar spindles and aneuploidy in cells, while inhibiting ERK1/2 did not cause defects during chromosomal events such as the spindle assembly checkpoint (SAC) and mitotic exit 49. The role of this phosphatase in the formation of multipolar spindles in cancer cells was recently investigated 50. In early mitotic mammalian cells, both VHR and ERK1/2 localized to the spindle apparatus 17,51, and transient VHR inhibition promoted the formation of multipolar spindles in human mitotic cells 50. These studies also demonstrated that depleting ERK1/2 activity but not JNK restored the multipolarity induced by a lack of VHR and that overexpressing VHR reduced ERK1/2 phosphorylation by reversing multipolar spindles. These results suggest that the VHR-mediated regulation of ERK1/2 plays multiple roles in genomic stability 50.

Many of the nuclear events that control genomic stability depend on high concentrations of proteins and/or rapid cytoplasm-nucleoplasm translocation. Interestingly, VHR is highly enriched in the nucleus of various cell lines 17,26,38,52, especially after genotoxic stress 26. This may indicate that this phosphatase has other substrates or that it has additional roles in maintaining genomic stability that could be directly or indirectly related to MAPK functions or other substrates. In fact, recent studies using bioinformatics approaches and validation analyses have suggested that novel VHR substrates are involved in genomic stability 26. In one study, the authors showed that VHR strongly co-localized with phospho-H2AX (Ser139) in cells exposed to radiation-induced DNA damage. They applied a bioinformatics analysis approach to identify human nuclear proteins that could be putative VHR substrates. Biochemical validation techniques were performed, resulting in some very promising targets, such as pATM (S1981), pATR (S428), pBRCA1 (S1423), BRCA2, CENP-F, Cyclin A, NBS1, APE1, MRE11, RAD50, pCHK2 (T68), and pP53 (S15). These results support the hypothesis that VHR is involved in genomic maintenance and that its depletion decreases survival and proliferation in human tumor cell lines by increasing DNA damage and/or delaying or impairing DNA repair 52.

Previous authors have also employed mass spectrometry to identify novel VHR substrates under cellular genotoxic stress conditions 27. Among the proteins identified, half were involved in mechanisms that control DNA and RNA structures and functions. Based on the presence of phosphorylatable tyrosine residues and what is known of the biological processes they regulate, experimental validation studies performed using cellular and biochemical assays suggested that nucleophosmin (NPM), nucleolin (NCL), and heterogeneous ribonucleoprotein isoforms C1/C2 (hnRNP C1/C2) are very likely to be VHR substrates 27. These proteins are tyrosine-phosphorylated *in vivo* and *in vitro* and could therefore be potential targets of dephosphorylation by VHR, especially because they are involved in cell cycle regulation and genomic instability (DNA damage response and repair) processes 53-56. Thus, the phosphatase activity of VHR against these three proteins should be further investigated.

VHR also mediates other signaling events in circulatory system cells and blood-related diseases 57. The first such studies were performed in resting T cells, which constitutively express VHR. During resting, activating TCR did not induce the expression of this enzyme via positive-feedback mechanisms. In T cells, VHR dephosphorylated MAPKs (especially JNK), but the UV radiation-induced activation of p38 was not targeted by VHR phosphatase activity 16. In Jurkat T lymphocyte cells, VHR activity was regulated by ZAP-70, a tyrosine kinase that phosphorylates VHR at Tyr138 22. Basal ZAP-70 activity was sufficient to stimulate T cells, but under some circumstances, such as when IL-2 was used to stimulate TCR, ZAP-70 activity induced more VHR-Tyr138 at the T-cell pole. These mechanisms regulated TCR and induced signaling 22.

A TCR-related signaling pathway was also affected by VHR in IFN-β-stimulated T and B cells via a mechanism involving STAT5. STAT5 is phosphorylated by tyrosine kinase 2 (TYK2). The Src homology 2 domain (SH2) of STAT5 recognizes phospho-Tyr138 on VHR and brings these domains together so that VHR can dephosphorylate STAT5. However, in this signaling cascade, VHR does not regulate the activity (via dephosphorylation) of other upstream effectors of the pathway, such as Janus kinase 1 (JAK1) and TYK2 19.

Another study showed that silencing VHR in *Staphylococcus aureus*-infected human and murine macrophages increased the production of pro-inflammatory cytokines via NF-kB signaling. The phosphatase activity of VHR seemed to affect this inflammatory response via a negative feedback component, and the observed increase in cytokine levels may have been responsible for the hyper-responsiveness observed in the host immune system 58. In contrast to the pro-inflammatory activities of VHR, VHR-deficient mice displayed tolerance to LPS-induced endotoxic shock and polymicrobial septic shock. A massive increase in anti-inflammatory M2-like macrophages, decreased TNF production, and reduced ERK1/2 activity were observed in these animals. *In vivo*, at eighteen hours after endotoxic shock was induced by LPS, DUSP3^-/-^ mice stabilize their body temperature, while DUSP3^+/+^ mice remained hypothermic. However, VHR knockout did not significantly affect T and B lymphocytes, neutrophils, monocytes, or platelet counts 59.

Additional studies have shown that VHR involvement in the immune response to septic shock is dependent on sex hormones produced by the host animals. Knocking out VHR did not protect male mice from LPS-induced endotoxemia or septic shock, and the percentage of M2-like macrophages was lower in these mice than in VHR^-/-^ female mice. The body temperatures of all groups of mice were lower after LPS injection (except in VHR^-/-^ females), while resistance to sepsis was associated with the decreased activation of ERK1/2, PI3K, and AKT, suggesting phosphatase deficiency. These data demonstrate that in the absence of VHR, female sex hormones are very likely transcriptionally involved in the acquired resistance of VHR^-/-^ mice to LPS-induced lethality 60.

Another very recent and important report provided mechanistic clues about the roles that VHR plays in platelet aggregation 61,62. This phosphatase seems to be responsible for reducing the levels of tyrosine phosphorylation of the enzymes Syk and PLCγ2, which regulate signaling via collagen receptor glycoprotein VI (GPVI) and C-type lectin-like type II (CLEC-2) receptors to promote aggregation in a MAPK-independent manner. VHR knock-out mice exhibited deficiencies in thrombus formation, suggesting that VHR contributes to arterial thrombosis but is unnecessary for primary hemostasis 61,62. The drugs currently used in clinics to treat thrombosis usually cause unwanted side effects, such as increasing the risk of gastrointestinal toxicity, neutropenia, thrombocytopenia, and bleeding, in addition to increasing the incidence of arterial thrombosis. Altogether, these novel functions of VHR strongly support the notion that this enzyme is a potential target for the development of new therapies for platelet aggregation in circulatory system cells and diseases 62.

## VHR expression and patient survival

It has been shown that VHR gene expression and protein levels are constitutively high in many different cell lines and diverse tissues 38,39. The VHR protein is highly stable even under high-stress conditions, such as cellular genotoxic or oxidative pressure 26,52. VHR gene and protein expression levels are not dependent on feedback regulation, such as that associated with many other MKPs 1,5,8,63. However, most VHR-inhibited cell lines exhibit phenotypes that are clearly associated with cell cycle arrest, senescence, and reduced cell survival even though VHR knock-out mice display a normal phenotype and are free of apparent pathological features 17,19,60,61.

However, one of the key remaining questions is that of how VHR expression varies among patients carrying different types of cancer. By mining freely available databases containing gene expression information related to human cancers, it is possible to analyze, for example, correlations between patient survival times and VHR expression. Using this approach, we investigated the *VHR* gene at the BioProfiling.de 64,65 website, which provides excellent assays that can be used to examine the functions, interactions, and pathway relationships of a single gene or a list of genes or to perform clinical correlations to test a gene of interest as a biomarker or to analyze its gene synergy (SynTarget tool) with relation to cancer survival (PPISURV tool).

**Figure 1:**
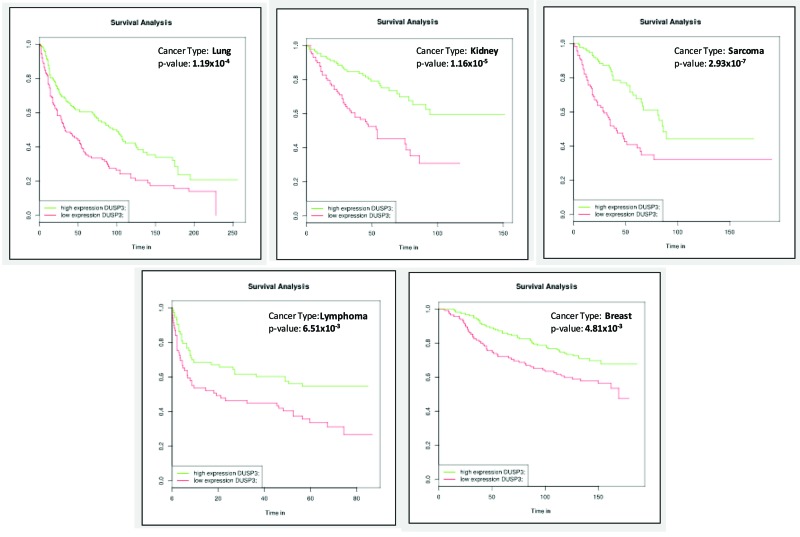
Survival curves generated using gene expression databases available at BioProfiling.de showing examples of different types of cancers in which VHR downregulation was correlated with a significant reduction in patient survival times.

VHR-containing gene sets are available for 13 different cancer types, including approximately 50 cancer subtypes or clinical variables, in this web tool. To illustrate the potential of this toolkit (and because these topics are very tightly correlated with previously published cellular experiments), we selected *VHR* gene expression and patient survival for our analysis 64,65. As shown in Figure 1, positive survival curves were observed when VHR was overexpressed in lung, kidney, sarcoma, lymphoma, and breast cancer. All five of these types of cancers have different GEO dataset IDs (mostly obtained from TCGA, https://cancergenome.nih.gov/) and clinical variables and are presented as data obtained from different numbers of samples (ranging from 240 to 290). The data showed that low VHR expression had a significantly negative effect on patient survival. These results, despite the fact that they are associative and predictive, when analyzed in primary and immortalized cell lines derived from the same tumor tissues, overall survival then was substantially lower in cells deficient in VHR than in control cells 17,19,52,66.

**Figure 2:**
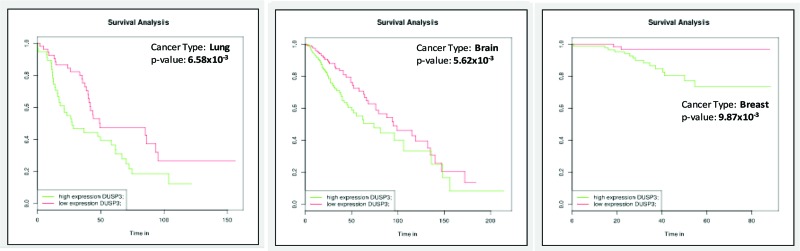
Survival curves generated using gene expression databases available at BioProfiling.de showing examples of different types of cancer in which VHR overexpression was correlated with a reduction in patient survival times.

However, as shown in Figure 2, VHR overexpression resulted in negative survival curves in lung, brain, and breast cancers. Although these cancers have different GEO dataset IDs, are associated with different clinical variables, and originated from a different numbers of samples (ranging from 115 to 450), high VHR expression had a statistically significant negative effect on survival in all three cancer types. These findings are in accordance with those presented in other reports in the literature showing that in some cell lines and tissues, VHR deficiency increases proliferation and survival 24,35, while VHR overexpression leads to cell death (usually by apoptosis).

An analysis of the correlations among the expression profiles of the *VHR* gene in various human cancers, patient survival times, and the cellular phenotypes related to VHR loss or gain of function (LOF or GOF) experiments showed that the biological functions of VHR seem to be tissue-specific. Additionally, VHR behaves both positively and negatively to regulate or mediate different types of human diseases. Therefore, the dual-specificity phosphatase VHR, similar to many other PTPs, has emerged as an attractive drug target for future clinical interventions 11,12,62,67.

General and non-specific PTP inhibitors have long been described in the literature, starting with sodium orthovanadate 68, α-bromo-4-(carboxymethoxy)acetophenone 69, phenylarsine oxide 70, and 4-hydroxy-3,3-dimethyl-2H-benz[g]indole-2,5(3H)-dione 71. Although these have been shown to be very potent inhibitory drugs, their low selectivity makes them poor candidates for clinical trials. Because of the diversity of substrates and biological functions attributed to VHR, which, as discussed in the previous sections, can be easily extended to animal studies and possibly even to a clinical setting, a great deal of effort has been made to identify specific VHR inhibitors. Once obtained, these compounds should be tested in animal models and humans. The results obtained in these studies will stimulate the development of therapies aimed at leveraging the inhibition of VHR and/or other phosphatases in diseases where the loss of VHR function may impair the proliferative capacity of diseased cells 67. It is especially important to facilitate the development of inhibitors that do not affect cellular processes in surrounding healthy tissues.

Some potent pharmacological inhibitors of PTPs/DUSPs are already in preparation for clinical trials, while others have received a growing amount of attention in the last few years. These include compounds that are usually obtained (identified, isolated and purified) from natural sources, such as plants, fungi, bacteria and seaweeds, and have high specificity for particular phosphatases 72. Additionally, very recently, an alternative to targeting DUSP enzymes at the transcriptional level was presented by research into omega 3 fatty acids ingestion. For example, docosahexaenoic acid (DHA) exerts a protective effect on mice brain development, and when it was added to the diet (in mice) as DHA-enriched fish-oil, it induced astroglial hyperactivation in a dose-dependent manner that was mediated by glial fibrillary acidic protein (GFAP) and dependent on increased MKP3 activity. This is the first evidence to show that natural compounds obtained from natural sources can have potential regulatory effects on PTPs/DUSPs during different cell biology and tissue developmental processes and suggest the possibility that they could be used to treat human pathologies 73.

## AUTHOR CONTRIBUTIONS

Russo LC and Farias JO wrote the first part of the review. Ferruzo PYM and Monteiro LF wrote the second part of this review. Forti FL wrote the third part and provided the figure data and also was the scientific mentor and supervisor of all authors.
